# CARer-ADministration of as-needed subcutaneous medication for breakthrough symptoms in homebased dying patients (CARiAD): study protocol for a UK-based open randomised pilot trial

**DOI:** 10.1186/s13063-019-3179-9

**Published:** 2019-02-07

**Authors:** Marlise Poolman, Jessica Roberts, Anthony Byrne, Paul Perkins, Zoe Hoare, Annmarie Nelson, Julia Hiscock, Dyfrig Hughes, Betty Foster, Julie O’Connor, Liz Reymond, Sue Healy, Rossela Roberts, Bee Wee, Penney Lewis, Rosalynde Johnstone, Sian Roberts, Emily Holmes, Stella Wright, Annie Hendry, Clare Wilkinson

**Affiliations:** 1 Bangor Institute for Health & Medical Research, Bangor University, Bangor, UK; 2 Betsi Cadwaladr University Health Board, Bangor, UK; 3 Marie Curie Research Centre, Cardiff University, Bangor, UK; 4 Cardiff and Vale University Health Board, Cardiff, UK; 5 Sue Ryder Leckhampton Court Hospice, Cheltenham, UK; 6 Centre for Health Economics and Medicines Evaluation, Bangor University, Bangor, UK; 7 North Wales Cancer Network Patient Forum, Bangor, UK; 8 Gloucestershire Hospitals NHS Foundation Trust, Cheltenham, UK; 9 Queensland Health Metro South Hospital Health Service, Brisbane, Australia; 10 University of Oxford, Oxford, UK; 11 The Dickson Poon School of Law, King’s College London, London, UK

**Keywords:** End-of-life care, Care of the dying, Palliative care, Symptom control, Carer administration, Randomised pilot trial

## Abstract

**Background:**

While the majority of seriously ill people wish to die at home, only half achieve this. The likelihood of someone dying at home often depends on the availability of able and willing lay carers to support them.

Dying people are usually unable to take oral medication. When top-up symptom relief medication is required, a clinician travels to the home to administer injectable medication, with attendant delays. The administration of subcutaneous injections by lay carers, though not widespread practice in the UK, has proven key in achieving home deaths in other countries.

Our aim is to determine if carer-administration of as-needed subcutaneous medication for four frequent breakthrough symptoms (pain, nausea, restlessness and noisy breathing) in home-based dying patients is feasible and acceptable in the UK.

**Methods:**

This paper describes a randomised pilot trial across three UK sites, with an embedded qualitative study. Dyads of adult patients/carers are eligible, where patients are in the last weeks of life and wish to die at home, and lay carers who are willing to be trained to give subcutaneous medication. Dyads who do not meet strict risk assessment criteria (including known history of substance abuse or carer ability to be trained to competency) will not be approached. Carers in the intervention arm will receive a manualised training package delivered by their local nursing team. Dyads in the control arm will receive usual care. The main outcomes of interest are feasibility, acceptability, recruitment rates, attrition and selection of the most appropriate outcome measures. Interviews with carers and healthcare professionals will explore attitudes to, experiences of and preferences for giving subcutaneous medication and experience of trial processes. The study has obtained full ethical approval.

**Discussion:**

This study will rehearse the procedures and logistics which will be undertaken in a future definitive randomised controlled trial and will inform the design of such a study. Findings will illuminate methodological and ethical issues pertaining to researching last days of life care.

The study is funded by the National Institute for Health Research (Health Technology Assessment [HTA] project 15/10/37).

**Trial registration:**

ISRCTN, ISRCTN 11211024. Registered on 27 September 2016.

**Electronic supplementary material:**

The online version of this article (10.1186/s13063-019-3179-9) contains supplementary material, which is available to authorized users.

## Background

### Introduction

Caring for the dying during their last few days of life, in a place of their preference, is an essential part of health and social care. The majority express a wish to die at home (79%); however, only half of those achieve this [1]. The likelihood of patients remaining at home often depends on availability of able and willing informal carers [2–4]. These carers take on numerous care tasks, often including the responsibility of assisting patients to have their oral as-needed medications. Extending the role of carers to include administering subcutaneous (SC) injections has proven to be key in achieving home death in other countries [5].

Pain, nausea/vomiting, restlessness/agitation and noisy breathing (rattle) are common symptoms in the dying [6, 7]. In addition to regular (background) medication, given via continuous SC infusion using a syringe pump, guidelines suggest using additional (‘as -needed’) medication for symptoms that ‘break through’ [8, 9]. As dying patients are commonly unable to take oral medication, as-needed medication is most often given as a SC injection by a healthcare professional (HCP) [8], usually a district nurse (DN) in the UK.

Medication for breakthrough symptoms is usually prescribed in advance (anticipatory prescribing) and kept in the patient’s home. Medication administration can be severely delayed by HCPs’ travel time to the home and/or the non-availability of anticipatory medication in the home. Delays happen even with dedicated out-of-hours (OOH) ‘rapid response’ nursing services for home-based dying patients. Our local audit revealed long waits. The median wait from call to OOH service for symptom control to as-needed medication administration by HCP was 86 min (mean = 99 min, range = 35–167 min), not including time from administration to onset of action or symptom control. Breakthrough pain is usually quick in onset with a median duration of 30 min [10]. Long waits mean that pain is often not adequately managed in the home setting, as shown in the National Survey of Bereaved People (VOICES) [1].

This project focuses on timely administration of as-needed medication for dying patients being cared for at home, particularly whether lay carer role-extension (to be trained to give as-needed SC injections) is feasible and acceptable in the UK.

### Rationale

Although carer administration of medication (including strong opioids) is lawful and practical [11], it is not currently part of usual care everywhere in the UK. This practice is much-needed: the national Palliative and End of Life Care Priority Setting Partnership (PeolcPSP) surveyed 1403 people including patients within the last years of life, current and bereaved carers, and HCPs on their unanswered questions about palliative and end-of-life care. They accorded highest priority to research into the provision of palliative care, including symptom management, outside of working hours to avoid crises and help patients to stay in their place of choice. The survey noted the information and training needs of carers and families to provide the best care for their loved one who is dying, including training for giving medicines at home [12]. Data from the PeolcPSP indicated that UK patients are being denied the opportunity to die at home due to lack of access to adequate symptom relief [13, 14].

Carers across the world embrace this as an option, as evidenced through the published literature as well as evidence from our Patient Public Involvement (PPI) group consultations. In Australia, the practice is well-established (> 30 years) and highly acceptable [5]. A manualised educational package and evidence-based guidelines are available.

Successful carer-administration of as-needed SC medication for breakthrough symptoms in a dying patient may:

- ▪ improve the experience (and thus increase the likelihood of a ‘good death’) for the patient who chooses to be at home by providing speedier symptom control and supporting their wish to die at home;
- ▪ empower lay carers through the personal fulfilment of having supported a patient’s wish to stay at home, increase satisfaction, and reduce anxiety and frustration related to poor symptom control;
- ▪ reduce inappropriate emergency (crisis) admissions due to uncontrolled symptoms and their associated costs [15, 16];
- ▪ free up community staff time to address other needs of patients and families, contributing to sustainability of services.

This practice appears acceptable and has become embedded in Australia. However, as per our rapid review, there are no randomised studies testing carer-administered non-oral medication in the last days of life for home-based patients anywhere else in the world.

Equipoise is emerging on this topic in the UK. Carer-administration of as-needed non-oral (including SC) medication for breakthrough symptoms in home-based dying patients is practised in a limited way in some areas in the UK and has been for a number of years. For this to be widely available to all carers who are considering supporting a loved one at home, it must be tested in a UK environment, with the support of an evidence-based carer education programme and resources. Not all family, carers or patients at home will want to be involved in this practice; the research will help to ascertain the proportion of those who are interested and how to train and support those who are willing.

#### How does the existing literature support this project?

A. Carers prioritise rapid symptom control and are willing and able to administer injectable drugs, including controlled drugs such as morphine: a narrative literature review of family carer perspectives on supporting a dying person at home illustrated the desire of families to provide immediate symptom relief [17]. Our review found that caregivers are willing to learn to overcome reservations about administering SC medications. The ability to alleviate their loved ones’ symptoms and support them to stay at home was of paramount importance.

B. There is an existing evidence-based education package and medication resources: a Brisbane group developed and evaluated an educational package [5, 18] and a randomised trial of who prepares the SC injections (carer, nurse or pharmacist) was completed [11]. In Singapore, a colour-coded pre-prepared ‘Comfort Care Kit’ is in use [19], with oral and non-oral as-needed medication for caregiver administration. A telephone survey of 49 family carers showed that 67% used the kit, all family members found it easy and 98% found it effective for symptom management. All except one patient died at home. In Canberra, the provision of an Emergency Medical Kit (including for use by lay carers) was largely viewed as an effective strategy in giving timely symptom control and preventing inpatient admissions [20].

C. There is growing UK evidence about the carer-role for patients in the last months/year of life, but there are few studies focusing on the last days of life (as reiterated by the Neuberger Review into the Liverpool Care Pathway) [21]. The evidence that is beginning to accumulate mostly focuses on patients who have capacity within the last year of life. UK/Australian research includes ‘Unpacking the home’ [22, 23]. The Cancer Carers Medication Management work [24], the SMARTE study [25] and IMPACCT [26]. Our project, in contrast, focuses on the last few days of life, where capacity is likely to be absent, with very different implications and issues for carer-administration.

#### Community receptivity

The UK is ready for testing this extended lay carer role:

- ▪ primary care teams and families are used to similar practices in other areas of medicine (insulin for diabetes, intravenous antibiotics for children with cystic fibrosis);
- ▪ the PeolcPSP report incorporated the views of 1403 people across the UK and placed great emphasis on empowerment of family carers and symptom management during the last days of life [12];
- ▪ the ‘Ambitions for Palliative and End of Life Care: a framework for local action’ was published in Sept 2015 [27]. It was jointly developed and published by the National Partnership for Palliative and End of Life Care (27 national organisations) and has widespread support, especially as the Partnership included the Patients’ Association and charities with large PPI groups. They identified eight foundations for the six ambitions; one of these foundations relates to ‘Involving, supporting and caring for those important to the dying person’, acknowledging their importance in the caring team. Each ambition has a set of building blocks – the one on ‘practical support’ in ambition 6 is particularly applicable to CARiAD as it calls for finding ‘new ways to give the practical support, information and training that enables families … to help’. There has been strong positive reception to the framework and many localities are using it to consider their local strategies. Its message about shared ownership and responsibility is particularly pertinent;
- ▪ in the UK, this practice is not widely accepted as usual care. However, over the past few years, we have identified a small number of geographically distinct sites where the practice occurs (< 10). Recently, the Lincolnshire project was showcased on national radio as part of a series of talks on dying [28, 29]. Since the conception of the CARiAD study, other areas have expressed interest in exploring the practice, including joining as a site of a future definitive trial.

#### Pressure on health and care services in the UK

HCPs in all three sites have been universally positive towards testing the intervention; if found to be beneficial, this could make their patients more comfortable and their jobs more manageable. In the longer term, this innovation could relieve some pressure on Emergency Departments by reducing inappropriate emergency (crisis) admissions due to uncontrolled symptoms [15, 16].

Pressure on DN time could also be relieved as extra visits (in addition to the daily check) to administer as-needed medication would decrease, contributing to sustainability of services.

### Phase 1 work

#### Expert stakeholder workshops

To inform the development of the intervention and specific processes at each site, three expert stakeholder workshops were conducted, one in each recruitment site. Half-day face-to face workshops were convened, based on the successful model used in the El-CID trial [30]. Each workshop had 10–15 participants representing patients, carers, general practitioners (GPs), DNs, pharmacists and specialist palliative care (SPC) clinicians. Two research team members facilitated, setting the context and background to the proposed intervention. Notes were kept which allowed a report of proceedings to be generated.

Participants discussed trial procedures, which were then developed based on the consensus reached. The issues covered were: Identification and risk assessment, and approach to potential participants; Consent; Prescription, supply and storage of drugs; Delivery of the intervention; Monitoring and accountability; Outcome measures collection; Post-bereavement interviews; and Ethical considerations.

Trial-specific materials were also developed to reflect the consensus reached: for HCPs – prescribing advice (relating to patients and carer in the intervention arm), competency checklist, risk assessment; for carers – Carer Diary, carer information booklet ‘Subcutaneous medication for breakthrough symptoms in the last days of life: a Guide for carers’, step-by-step guides.

### Study aims

Research Question: Is carer-administration of as-needed SC medication for breakthrough symptoms in homebased dying patients feasible and acceptable in the UK?

- P = Patients in the last days of life who are becoming unable to take their usual oral as-needed medication for breakthrough symptoms, being cared for at home, and their carers
- I = Carer-administration of as-needed SC medication for common breakthrough symptoms such as pain, restlessness/agitation, nausea/vomiting and noisy breathing/rattle, supported by tailored education
- C = Usual care (HCP-administration of as-needed SC medication)
- O = Main outcomes of interest: Feasibility and acceptability, recruitment, attrition, contamination

We will undertake a randomised pilot trial of carer-administered as-needed SC medication for common breakthrough symptoms in home-based dying patients versus usual care, with an embedded qualitative component, to inform the design of a future definitive phase 3 randomised controlled trial (RCT).

## Methods/Design

### Trial design and setting

The study will be a multicentre pilot RCT carried out in community settings in Gloucestershire, North Wales and the Vale of Glamorgan where patients are likely to die at home in accordance with their wishes. The three pilot study sites have been chosen as they are representative of the range of sites for a future definitive study.

The trial was funded by the Health Technology Assessment Programme of the National Institute for Health Research (National Health Service). It has received a favourable ethical opinion from the Wales 1 National Research Ethics Committee (REC reference: 17/WA/0208, IRAS project ID: 227970) and the Bangor University Research Ethics Committee. The UK Medicines and Healthcare products Regulatory Agency (MHRA) has advised that this pilot RCT is not a Clinical Trial of an Investigational Medicinal Product (CTIMP). The study is registered on the International Standard Randomised Controlled Trials Number (ISRCTN) registry (ISRCTN11211024). Approval was granted from the Research and Development departments of all three sites. SPIRIT 2013 recommendations and CONSORT 2010 statements (including those specific to randomised pilot and feasibility trials) guided protocol development [31, 32]. The current version of the protocol can be accessed via the National Institute for Health Research (NIHR) Journals Library [33].

#### Inclusion/exclusion criteria for participants and recruitment

##### Inclusion criteria

Dyads of

- ▪ an adult (aged ≥ 18 years) patient in the last weeks of life, who is likely to lose the oral route for medication and who has expressed a preference to die at home; and
- ▪ their adult (unpaid) lay/family carer, who is willing to have this extended role and have SC-injection training.

Prognostication is reliant on the professional judgement of, and agreement within, the attending HCP team. There is an assumption that the carer will spend a significant amount of time with the patient. While Australian experience indicates that one lay person generally takes a lead role in this practice, where there is more than one suitable carer, we will ask the patient to identify which carer they would like to be included in the study.

##### Exclusion criteria

Patients who have only paid/formal care or with previously known adverse reactions to the ‘usual’ as-needed medications will be excluded. Patients or lay carers who have not met the risk assessment criteria (see below) will not be approached for consent.

##### Patient identification

Patient / carer dyads will be identified through the hospice, SPC service or DN team. When a patient is perceived by the HCP team to be in the last weeks of life and they have expressed a wish to be cared for and die at home, they will be screened for approach.

##### Screening

To be eligible, dyads must satisfy the risk assessment criteria. A risk assessment screening tool has been refined for CARiAD, based on existing self-medication tools [34]. Risk assessment is based on clinical knowledge and judgement of the situation and will take into account several factors, including:

- ▪ the carer’s mental state, vision and physical condition;
- ▪ the dyad’s attitudes to medicines and willingness to engage with the healthcare team;
- ▪ relational issues including concerns about burden;
- ▪ history of substance misuse in the family.

The risk assessment will be conducted by the healthcare team involved in the patient’s care. If a dyad does not satisfy the risk assessment criteria, they will not be approached.

##### Approach

The patient will be approached with written material by a member of their healthcare team. The initial patient approach will be done separately from the carer, unless otherwise requested by the patient and if the attending HCP deems this appropriate, i.e. there is no perceived risk of patient–carer coercion. As the project involves sites in Wales, the Participant Information Sheets and consent forms will be translated into Welsh for the Welsh centres and offered bilingually to comply with the Welsh Language Act 1993. Dyads will be given as much time as they need to consider the information sheets and discuss with family, friends or the healthcare team until they decide whether to take part. They will be told that they can refuse participation without giving reasons.

##### Informed consent

A researcher will seek advance consent separately from both the patient and their lay carer at a time judged to be suitable by the attending HCP. This gives the patient and carer as much time as they need to understand the nature of the research, ask questions and make their feelings clear on trial participation [35, 36].

If the patient is unable to consent, or once they lose capacity after they have previously given consent, the assent of a Personal Consultee will be sought (as required by the Mental Capacity Act 2005) to the patient’s participation in the trial [35, 36]. As the risk assessment will exclude dyads where there are concerns about relational issues between patient and carer, the carer can act as Personal Consultee.

If the carer does not wish to act as the Personal Consultee and there is no additional family member or close friend to take on this role, we will appoint a Nominated Consultee (e.g. a HCP not associated with the research) who will act for all patients in this situation in the trial.

##### Randomisation

Once the dyad has consented and baseline data has been completed, the dyad will be randomised to one of the trial arms. Secure online randomisation hosted by the North Wales Organisation for Randomised Trials in Health (NWORTH) Clinical Trials Unit will be performed by the researcher who has obtained consent. The system will use a dynamic adaptive method of randomisation stratifying for recruitment centre and diagnosis (cancer/non-cancer) [37]. Confirmation of allocation will be sent only to those members of study staff who need to be aware of the result.

##### Blinding

CARiAD is an open trial where blinded outcome assessment is not feasible; therefore, it is important that outcomes are as robust as possible in light of the lack of blinding. Outcome assessors will be experienced research nurses. Data entry will be completed unblinded; the trial statistician providing data analysis will be the only individual blinded to randomisation allocation.

##### Withdrawal criteria

Participants remain free to withdraw at any time from the trial without giving reasons and without prejudicing their further treatment. This will be made clear to all potential participants at the time they consent to participation and throughout their time in the trial. Non-completion of the follow-up questionnaires will not constitute formal withdrawal from the trial; unless the participant requests withdrawal of their data completely, it may be used to impute values for the analysis. The risk assessment will be reviewed at intervals based on HCP judgement and if the criteria are not met the dyad will be withdrawn from the trial.

### Interventions

Training of carers in the intervention arm will be supported by a manualised training package based on the Australian package ‘Caring Safely at Home’ [5]. Lay carers will receive training on: common symptoms that may occur in the last days of life and how to assess if their loved one needs medication for a particular symptom; how to prepare (draw up) medication and dispose of sharps (glass ampoules and drawing up needles); how to administer SC medication by needle-less technique (utilising a ‘butterfly’ SC catheter); how to assess the effect of the medication; and support available, including primary care team as well as dedicated 24/7 SPC support. If a symptom occurs for which medication is deemed necessary (either as expressed by the patient, if able, or as assessed by the carer), the carer can use the training outlined above to administer the appropriate medication.

#### Medication regimens

Guidelines for anticipatory prescribing for last days of life care are in place across the UK [38, 39]. They cover common symptoms in the dying phase: pain; nausea and/or vomiting; restlessness/agitation; and noisy breathing/rattle. CARiAD recruitment sites will be advised to follow usual prescribing practice. For patients in the intervention arm only, prescribers will be provided with specific additional advice, including instructions not to prescribe dose ranges/steps, and that dose changes can only be made after a face-to-face assessment (and not remotely, i.e. over the telephone).

#### Care pathways

The usual care arm has an unchanged care pathway for dealing with breakthrough symptoms at home for a dying patient, with usual palliative care and DNs administering as-needed SC medication.

‘Usual routes’ for support in each recruitment area are different. For some areas, there is direct access to a 24/7 SPC advice line for carers in addition to support from the patient’s primary care team within or out-of-hours. In other areas support for the carer will be via their primary care team, while the GPs and DNs can call on advice from SPC clinicians.

In the intervention arm, carers will be trained to administer as-needed SC medication, although they will not be obliged to do this. If the carer needs the support of a HCP, either because they would feel more confident having a HCP present when they administer medication or they wish the HCP to assess and give medication, they can obtain it via the usual routes in their area. If the carer has reached the limit of the number of injections which can be given in 24 h (maximum three injections for each indication per 24 h period unless the prescribing clinician advised a maximum of fewer than three), they will be asked to contact a HCP as review is indicated. Usual routes for support might include DN team, GP, GP/DN out-of-hours, Hospice at Home team or a hospice advice line. The use of such support will be captured in carer diaries (see ‘Study procedures’).

#### HCP training requirements

In order for nurses to train carers, they will themselves receive training on: the standardised manualised education package (adapted from the Australian work); the legal framework (see Additional file 1); guidelines for medication handling and administration in a community setting; and on trial-specific materials and processes.

### Study procedures

For an overview of study assessments, see Table 1.

**Table 1:** Overview of study assessments

Assessments		Screening	Baseline	Last days of patient’s life	After patient’s death
Eligibility		X	X	X	
Informed consent			X	X	
Demographic information			X		
Medical history			X		
Concomitant medications			X		
Symptom control	Symptom scores			X	
	Overall symptom burden				X
	Time to symptom relief			X	X
Safety	Risk assessment	X			
	Competency Checklist		X	X	
	Significant Event reporting			X	
	Evaluation of training package				X
Impact on carer	Self-efficacy		X	X	X
	Confidence			X	
	Acceptability				X
Health Economic outcomes	Impact on carers		X		X
	Discrete Choice Experiment attribute selection				X

The main outcomes of interest will be those appropriate to a pilot trial, including feasibility, acceptability, recruitment rates, attrition and selection of the most appropriate outcome measures. Outcomes will be measured for patients, their lay carers and HCPs. System barriers will also be noted. These measurements will be made at baseline, on a daily basis for symptom control and lay carer confidence, at 6–8 weeks after bereavement and at 2–4 months for a sub-sample (carer interviews).

Recruitment measurements are: the number of eligible patients who fulfil the inclusion criteria and are willing to be randomised expressed as a percentage of the numbers screened; the number who withdraw after baseline assessment and randomisation; the number who complete the various outcome measurements at baseline and at later time points; and reasons for any non-completion.

Patient measurements are: baseline information (including demographic information, medical history, capacity assessment, preferred place of care in the last days of life, current drug management) and a daily Carer Diary during the study related to the presence and treatment of breakthrough symptoms (for use in both study arms). Data points in the diary will include: initial time breakthrough symptom triggered perceived need for an additional SC dose; whether noted by patient or lay carer; medication and dose, and time given; reason for medication (pain, nausea, restlessness, noisy breathing); symptom score before and 30 min after medication administration; and when symptom control/reduction of symptom to acceptable level was achieved. Hospital or hospice admissions during last illness and actual place of death will also be recorded.

Carer measurements are: demographic information at baseline; Quality of Life in Life Threatening Illness – Family Carer Version (QOLLTI-F) (at baseline, after the first as-needed SC medication, then every 48 h until the patient’s death); whether HCP support was sought; Carer Experience Scale at baseline and after bereavement; Family Memorial Symptom Assessment Score – General Distress Index (MSAS-GDI) at 6-8 weeks' post-bereavement visit; and qualitative interviews for a sub-sample at 2–4 months after bereavement. Specific to the intervention arm, confidence (in administering injection) and competence at intervals after training will be recorded.

HCP measurements are: baseline measurements of attending team structure; primary prescriber; carer trainer; and evaluation of the training package.

#### Safety

The CARiAD project contains a number of safety outcome measures at different stages of the clinical journey taken by patients, carers and HCPs. Safety outcome measures include the risk assessment, competency checklist and Significant Event reporting. Significant Event reporting will include the following: the appropriateness of administration (is administration accompanied by evidence of need?); proportionality (has the correct dose been administered?); side effects both anticipated and not anticipated; drug accountability (do stocks tally?); and carer events (e.g. distress, needle stick injury, accidental or purposeful self-administration).

An adverse event (AE) is defined as any untoward medical occurrence in a trial participant (either patient of carer) and includes incidents that are not necessarily caused by or related to the trial. A serious adverse event (SAE) is any untoward occurrence that results in death, is life-threatening, requires inpatient hospitalisation or prolongation of existing hospitalisation, results in persistent or significant disability/incapacity or is otherwise medically significant.

All AEs and SAEs will be captured via Significant Event form. SAEs must be reported to the Principal Investigator (PI) and Sponsor within 24 h. As this is a study in patients who are terminally ill, death is an expected outcome. It will be recorded and reported to the sponsor, but will not be considered a SAE if, in the opinion of the PI, it was a natural conclusion to a patient’s terminal illness. Due to the nature of the study, events of death will not require immediate reporting to the Data Monitoring and Ethics Committee (DMEC).

### Exploratory endpoints/outcomes for a future definitive trial

The most likely candidates for primary outcome measures for a future definitive trial are: MSAS-GDI (a measure of overall symptom burden/distress in the last seven days of life) [10, 40–42] and QOLLTI-F (a measure of quality of life of carers looking after someone with a life-threatening illness, incorporating elements of control and self-efficacy) [43].

In addition, we will measure carer confidence using a 5-point Likert scale (where the carer is asked after administration of every as-needed SC injection to rate their level of confidence in administering this injection, 1 = not at all confident, 5 = very confident) and probe carer experience during qualitative interviews.

Criteria for assessing feasibility as primary outcome measure: all outcome measures will be assessed on the same criteria (applicability, acceptability and level of completeness) for consistency. Once the feasibility of the outcomes is established, the design of the definitive trial will be confirmed. The potential suitability of the following secondary outcomes will be considered: ‘Time to symptom relief’ and Carer Experience Scale [44–46].

### Embedded qualitative study

The aim of the embedded qualitative component is to inform the design and assess the feasibility of a phase 3 trial of carer-administered medication. The study will collect interview data from HCPs and carers to:

- ▪ assess clinical willingness to randomise patients for a future full RCT;
- ▪ understand the experience of randomisation between intervention and control, to identify relevant patient-centred outcomes for a phase 3 trial and to consider time points for assessment.

The qualitative study aims to include interviews with non-consenters to the trial, as well as in-depth qualitative exploration of carer and HCP acceptability of carer-administered SC medication, e.g. strong opioids, anti-emetics, sedatives. The study will use a phenomenological and pragmatic approach to understand the meaning that carer-administration of injectable strong opioids and other as-needed medication has for bereaved carers and HCPs and the practicalities involved.

#### Sample

Face-to-face qualitative interviews across the three recruitment sites will be conducted with:

- ▪ 6–10 carers who have experience of supporting a patient in the intervention arm;
- ▪ 6–10 carers who have experience of supporting a patient receiving usual care;
- ▪ 6–10 carers who declined to be randomised to the trial. For carers in all three groups, sampling criteria will include gender and rurality;
- ▪ up to 30 HCPs – to include prescribers (e.g. GPs and ANPs), administering HCPs (e.g. DNs) and SPC clinicians. Sampling criteria will include years since qualification, experience of supporting home deaths and practice characteristics.

#### Consent

Carers declining to take part in the trial will be approached upon declining and invited to participate in an interview about the reasons why they chose not to participate. They will be given a separate information sheet for this.

#### Data gathering

Interview topic coverage was informed by PPI input, the systematic review and the expert consensus workshop. Attitudes to and experiences of having administered medication including emotional, ethical and practical reflections will be explored, as will issues relating to trial recruitment and feasibility (supply and storage of medication, success of training and perceived competence of carer once trained, choice and recording of the primary outcome). Carers will be interviewed approximately 2–4 months after bereavement (as suggested by usual clinical follow-up and current literature) [1, 47–49].

Interviews will be face-to-face at carers’ homes or alternative preference, or possibly by telephone, lasting 30–60 min. The interviews with carers who declined to be randomised to the trial will be shorter, lasting 15–20 min. HCP interviews will be by telephone and last around 30 min. All interviews will be audio recorded, transcribed verbatim and the carer interviews will be managed using NVivo. Participants will be asked to consent to publication of anonymised quotes.

#### Analysis

The analytic frameworks are selected to understand the meaning that carer-administration of injectable strong opioids and other as-needed medication has for bereaved carers and HCPs. Carer interviews will be analysed using Interpretative Phenomenological Analysis [IPA] to allow a deeper, inductive analysis of the data in the context of carers’ and patients’ daily lives and values [50]. This methodology focuses on the subjective experience of participants, as interpreted by the researcher. HCP interviews will be analysed using Framework Analysis with a deductive approach [51]. Framework analysis is commonly used in healthcare and is more appropriate for examining the specific aims and objectives of the HCPs. The data will be summarised thematically and displayed on a matrix linking to the original data.

### Identification of attributes for a future Discrete Choice Experiment (DCE)

We have identified the need to determine carers’ preferences for HCP versus own administration of medication to patients, using a DCE. The preferences of carers towards administering SC medications will have a bearing on their willingness to adopt this practice and the effectiveness of carer-administered medication.

While the DCE (aiming to ascertain carers’ preferences for their administration of SC medications) will be conducted as part of a future main study, the preparatory work required to identify relevant attributes and levels will be done as part of the embedded qualitative study component of the randomised pilot study. This will be done with each of the three carer groups in the second part of the interviews and will take up to 20 min. Attributes may feasibly include cost, time, perceived competency, confidence and potential risks. The process of attribute development will be informed by best practice [52].

The use of interviews for the determination of DCE attributes enables a greater opportunity for in-depth exploration of particular issues and concepts than would otherwise be possible in focus groups (which are more common in DCE development). Individual interviews are also better suited to discussions concerning sensitive topics. Within the first five interviews in each group, carers will be presented with a range of attributes, identified by the research team as being likely to affect carers’ choice for own versus HCP administration of SC medications. Interviewees will have an opportunity to add other factors of their own choosing to the list and be asked to identify and rank attributes in order of importance. Thereafter, we will use the interviews to pilot the presentation of the highest ranked attributes. The ordinal ranking across each group will be determined and those ranked highest will be taken forward for DCE development. We have successfully implemented this method in previous DCEs [53] and it is consistent with the reductiveness approach of attribute development [52].

We will also pilot the Carer Experience Scale as a means to estimate carer utility [46]. The index values derived from this scale offer a preference-based approach to incorporate the effects on carers in economic evaluation, focusing on care (rather than health)-related quality of life.

### Statistical considerations

#### Sample size

A fully justified sample size is not required; size has been justified by estimating what a future definitive RCT will need. Assuming an important difference of 0.4 (SD=1) on the Family MSAS-GDI a sample of about 216 is required to achieve 90% power to detect a difference of this size with a significance level of 0.05 using a two-sided test. Equivalently a sample of about 550 would be required to detect a difference of 0.5 points (SD=2) using the QOLLTI-F.

**Fig. 1:**
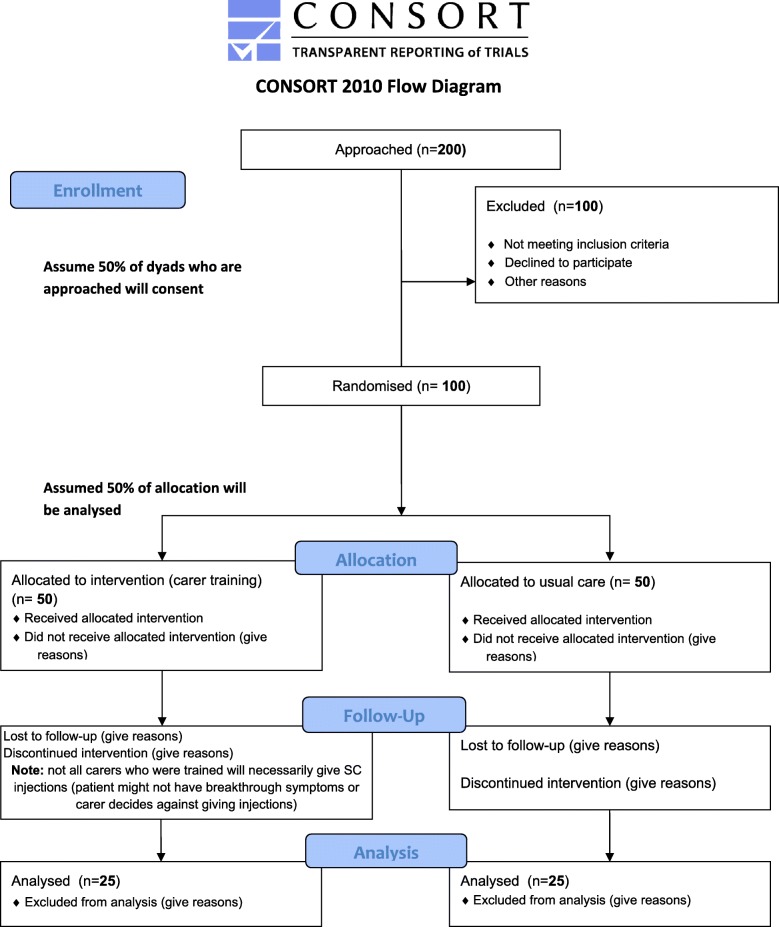
Trial *flowchart*

Using the larger of these estimates for the feasibility trial, we will assume about 9% of the main trial size, to give an 80% confidence interval to exclude a clinically important difference, requires ~ 25 in each group [54]. Sim and Lewis recommend a sample of about 50–55 to ensure robust estimates of the variance [55]. Using estimates of dropouts, we predict we need to approach 200 potential participants to achieve 100 randomised participants, with 50 completers. (‘Completer’ is defined as a dyad who completed all the study measures from baseline to follow-up at 6–8 weeks after bereavement.) We will therefore need to approach 5.5 dyads per month from each of the three sites and randomise 2.7 dyads per months from each of the three sites to meet our recruitment target. Assuming we will recruit equally between the three areas, we need to approach 66, randomise 33–34 and have 16–17 available per area for analysis (see Fig. 1: Trial flowchart).

As per the 2013 Office of National Statistics data described earlier, we know that 8.6% of all deaths are home deaths due to neoplasms in those aged > 15 years [1]. Deaths due to neoplasms are seen as a useful proxy for expected deaths. Therefore, the three recruitment areas have the following numbers available per annum: North Wales = 653; Gloucestershire = 517; and Vale of Glamorgan = 349.

#### Statistical analysis

Primary analysis will be concentrated on the feasibility metrics and adherence outcomes based on the thresholds defined in Table 2. There will be limited preliminary analysis of intervention outcomes. Point and 95% confidence interval estimates will be calculated and used to estimate variability and direction of effect to further inform the sample size calculation for a definitive study.

**Table 2:** Objectives, action plan and criteria for progression to a full trial

Objectives	Action plan	Threshold for progression to definitive RCT
1 To refine the assessment and outcome measures to be used in any potential RCT	Qualitative feedback will be collected from participants 2–4 months after the intervention, regarding the acceptability of the measures and will evaluate whether all of the intended information was captured	
2 To evaluate the acceptability of the manualised intervention (and potentially refine)	An initial workshop with the Australian team was held (Nov 2015). Expert consensus workshop discussions led to refined trial processes, education package and resources [30] A detailed process is described in the study protocol clarifying the legal and regulatory framework for the practice	In the feasibility study the simplest method is for lay carers to draw up medications only in immediate form; a full trial would be more appropriate if able to extend this to advance preparation and labelling
3 To evaluate the recruitment process	Referral sites and referral sources Where participants heard about the study Number and speed of referrals received and time elapsed between initial contact made with the study team (for information and consent form)	In the feasibility we have assumed 50% recruitment – we would say a full trial is not possible if recruitment falls < 30%
4 To estimate participant retention rate for the full RCT	Retention rates will inform the refinement of the sample size calculation for any potential subsequent RCT Participant engagement will be monitored throughout the pilot trial	In the feasibility we have assumed 50% retention – we would say a full trial is not possible if retention falls < 40%
5 To test the assessment and outcome measures for suitability, relevant change factors and acceptability to participants	Data from the assessment process will be compared against raw data from the outcome measures to assess the outcome measures sensitivity to identifying participant change	
6 To identify acceptability and collection of relevant data to inform the data collection and analysis plan for implementation in the subsequent RCT	A review will be completed of each outcome measure of levels of missing data and stability to ensure that the information collected will allow any future main analysis to be feasible and appropriate. Amendments can be suggested where appropriate to amend data collection for any potential future trial. The data available will also inform the details for the analysis plan of any potential full trial	Carer Diary data items successfully completed (70%) Family MSAS-GDI successfully completed at bereavement visit (70%) QOLLTI-F successfully completed at 48-h intervals (70%)

Summary statistics of all outcomes will be used to inform the approximate models of analysis that would be used in a full trial. Models will be specified once the data is better understood through the feasibility trial (e.g. numbers of episodes where as-needed medication used, proportion of participants that never required as-needed medication). A preliminary analysis of the outcomes will be completed using an intention-to-treat approach. All analysis undertaken will be prespecified in a statistical analysis plan that will be written and agreed before data collection is completed.

As this is a feasibility trial, there will be no imputation of missing data. Missing data will be considered as a criterion for assessing the suitability of measures. Descriptive statistics will be produced for each of the outcome measures, to evaluate the appropriateness of the measures for inclusion in a definitive RCT.

#### Progression to full trial

Clear progression rules are defined to determine whether an application for a future substantive trial powered to study effectiveness and cost-effectiveness should proceed. Our progression rules will relate to the following measures; which we considered important to feasibility:

- reaching our target (16–17) for the number of patients recruited per site.

We have also established clear assessment criteria for establishing the acceptability of the potential primary outcome measures.

The table below summarises the objectives, action plan and criteria for progression to a full trial.

### Governance

Trial governance procedures adhere to the NIHR guidelines and include a Trial Management Group (TMG), an independent Trial Steering Committee (TSC) and an independent DMEC. SAEs will be reported to the TSC and DMEC in line with NIHR guidance [56].

#### Trial Steering Committee (TSC)

The TSC nominees have been reviewed and appointed as members by the NIHR Programme Director. The independent members include a Chair, statistician, primary and palliative care clinicians and two public contributors. All TSC meetings have a minimum of 75% majority of independent members. TSC responsibilities include [56]:

- ▪ providing advice, through its Chair, to the Trial Funder, the Trial Sponsor, the Chief Investigators, the Host Institution and the Contractor on all appropriate aspects of the project;
- ▪ concentrating on progress of the trial, adherence to the protocol, patient safety and the consideration of new information of relevance to the research question;
- ▪ ensuring that the rights, safety and wellbeing of the participants are the most important considerations and should prevail over the interests of science and society;
- ▪ ensuring appropriate ethical and other approvals are obtained in line with the project plan;
- ▪ agreeing proposals for substantial protocol amendments and provide advice to the sponsor and funder regarding approvals of such amendments.

#### Data Monitoring and Ethics Committee

The DMEC nominees have been reviewed and appointed as members by the NIHR Programme Director. All members are independent and include a Chair, statistician, health economist and palliative care clinician. DMEC responsibilities include [56]:

- ▪ monitoring the unblinded comparative data and make recommendations to the TSC on whether there are any ethical or safety reasons why the trial should not continue;
- ▪ considering the need for any interim analysis advising the TSC regarding the release of data and/or information;
- ▪ providing, at the request of the Project Funder, a confidential interim or futility analysis if serious concerns are raised about the viability of the study or if the research team are requesting significant extensions.

To conform to the Data Protection Act 2018 and the General Data Protection Regulation (GDPR) (Regulation [EU] 2016/679), all data will be anonymised and stored securely. No published material will contain patient identifying information.

##### Peer review

This protocol has had high-quality (independent, expert and proportionate) peer review through the NIHR HTA funding application process. The independent members of the TSC and DMEC will provide an element of continuous peer review.

Bangor University is sponsoring the study, and Professor Chris Burton (Head of School, School of Healthcare Sciences, c.burton@bangor.ac.uk) is acting for and on behalf of the Study Sponsor. Sponsor responsibilities include:

- ▪ taking responsibility for putting and keeping in place arrangements to initiate, manage and fund the study;
- ▪ confirming that everything is ready for the research to begin;
- ▪ satisfying itself that the research protocol, research team and research environment have met the appropriate scientific quality assurance standards;
- ▪ satisfying itself that the study has ethical approval before relevant activity begins;
- ▪ allocating responsibilities for the management, monitoring and reporting of the research;
- ▪ ensuring that appropriate arrangements are in place to approve any modifications to the design, obtaining any regulatory authority required, implementing such modifications and making them known;
- ▪ satisfying itself that arrangements are kept in place for good practice in conducting the study and for monitoring and reporting, including prompt reporting of suspected unexpected SAEs.

### Quality assurance and quality control

#### Monitoring, audit and inspection

A Trial Monitoring Plan will be developed and agreed by the TMG and TSC based on the trial risk assessment. Site monitoring will be done by performing site visits (at least once per site, with a specific focus on consent recording and handling of data and site files) as well as remotely by exploring the trial dataset.

The sites will be expected to assist the sponsor in monitoring the study. These may include hosting site visits, providing information for remote monitoring or putting procedures in place to monitor the study internally. Monitoring will be conducted across all sites and will include a focus on enrolment rates, numbers of withdrawals and numbers of reported AEs.

Responsibilities for monitoring will be defined and documented in the Trial Monitoring Plan.

### Data handling

Procedures are in place to protect participant confidentiality before, during and after the trial.

#### Data collection tools and source document identification

Source data will be captured on paper at the relevant time points. A study-specific MACRO database will be developed to allow researchers to enter data online. MACRO allows controlled access to the data by all centres and stores a full audit trail. The electronic data captured in the MACRO database will be stored on servers maintained by Bangor University and will be subject to the university IT disaster recovery procedures.

#### Access to data and data management

Paper data at sites will be stored in locked filing cabinets separately from identifiable participant data. Access to the MACRO site will be secure and password-controlled.

Access to MACRO will be defined on two different levels—access to input (researchers at sites) and access to full dataset—which will be limited to those core team members involved in data and trial management.

A detailed data management plan will include the definition of the data quality checks that will be performed on the data throughout the life course of the trial. These will include source data validation, random data checks and timelines for data entry.

#### Access to the final trial dataset

The trial statisticians will have full access to the dataset. The Chief Investigators (CIs) and trial manager will have access to the full dataset after the analysis has been completed. The DMEC will have access to the full dataset as required. The TSC will have access to the full dataset before the individual sites having access.

#### Data sharing

During the course of the trial, datasets may be requested from the trial team. A data request form will form part of the data management plan and will document the approval and retrieval process for datasets during the conduct of the trial. All requests will have to be approved by the CIs. All data requests and datasets issued will be retained for completeness.

#### Data archiving

Archiving of trial documents will be authorised by the Sponsor following submission of the end of study report. As per the sponsor’s research data management policy, research data and records will be retained ‘for as long as they are of continuing value to the researcher and the wider research community, and as long as specified by research funder, patent law, legislative and other regulatory requirements. The minimum institutional retention period for research data and records is five (5) years after publication or public release of the work of the research, unless required by the funder to retain for longer’ [57].

In line with legal requirements, trial documents will be archived centrally at a secure facility with appropriate environmental controls and adequate protection from fire, flood and unauthorised access. Archived material will be stored in tamper-proof archive boxes that are clearly labelled. Electronic archiving will be provided by the sponsor for post-project deposit and retention of data. Destruction of essential documents will require authorisation from the Sponsor.

### Publication policy

#### Dissemination plan

The results of the study will be first reported to trial collaborators. The main report will be drafted and agreed by the trial coordinating team and the final version will be agreed by the HTA before submission for publication, on behalf of the collaboration.

The study findings will be disseminated through publication in highly cited and open-access peer-reviewed journals and submissions to national and international conferences. In addition, dissemination of our work to clinical and academic colleagues will be via professional societies, newsletters, existing networks and professional websites. Relevant NHS organisations and healthcare providers, e.g. Clinical Commissioning Groups and National Institute for Health and Care Excellence (NICE), will be informed of the study outcomes.

All carer participants, if they so wish, will be sent an accessible summary of the findings from the study within six months of study completion. The same summary will be made available to public/patient forums to inform patient groups across the area.

It is expected that the TMG will ensure a high level of awareness of our work in the relevant media while exploring the use of social media to disseminate outcomes, encourage public/patient involvement and promote future research to improve patient care at the end of life.

#### Authorship eligibility

Authorship (individually named or group) on the final trial report and manuscripts submitted for publication will be in accordance with the authorship criteria defined by the International Committee of Medical Journal Editors [58].

### Indemnity

Bangor University has appropriate Clinical Trials Indemnity and Professional Indemnity insurance in place that will cover members of the research team to conduct the research as per protocol. Health and Care Research Wales staff has NHS contracts and will be responsible to ensure that their work is appropriately insured. NHS staff working with patients involved in the intervention will not be expected to do anything that is not covered by their contracts and will remain covered by the NHS insurance arrangements.

## Discussion

Empowering lay carers to support a loved one’s wish to die at home is an important part of care in the last days of life. Role extension for lay carers allowing them the option to give SC PRN injections is already practised and valued in other countries. It is important and timely to study this in the UK setting. In order to design a definitive study of the clinical and cost-effectiveness of carer administration in this context, rehearsal of the study procedures and logistics is required. The study we describe will fulfil this aim and will provide an exemplar for conducting RCTs in the last days of life by contributing to the emerging methodological development of palliative care research.

Specific design considerations resulted in the decision to propose a stand-alone (external) randomised pilot trial. These include:

- ▪ the current UK context (post-Shipman, post-Liverpool Care of the Dying Pathway and with the ongoing euthanasia public debate), calling for careful attention to its impact on consent mechanisms and attitudes of carers, patients and HCPs to this innovation;
- ▪ the lack of clear UK-wide guidance on carer-administration of as-needed SC medication to dying home-based patients: though the practice is lawful, current guidance is not detailed nor specific enough for wide adoption;
- ▪ the lack of a clear and widely accepted training package for lay carers, adapted for the UK context;
- ▪ the uncertainty about the primary outcome measure for a definitive trial.

These are unpredictable barriers until the re-worked Australian manualised intervention is introduced and trial processes are tested. If the intervention is proven feasible and acceptable, we anticipate a phase of ensuring new guidance is developed and put in place at national level in UK health systems to enable the practice before rolling out a full trial quickly. We have demonstrated a clear path towards a definitive RCT as per the Medical Research Council Framework for the evaluation of complex interventions principles, further informed by the Methods of Researching End of Life Care guidance [59–61].

The CARiAD team is aware of the sensitivities surrounding, and ethical and methodological challenges associated with, researching last days of life care. With this in mind, study processes were carefully developed with strong public contribution.

### Public contribution

Our team is committed to meaningful involvement of patient representatives. Two service users are co-applicants. Insights gained from their experiences of giving injections to dying loved ones at home were crucial in designing the project and they have offered to be involved at all stages of its development. Their involvement will be fundamental in disseminating the research results to patients, carers and healthcare professionals. Two additional groups of bereaved carers have been consulted and their suggestions on consent mechanisms, drug safety, training and ongoing support have been incorporated into the study design. The recruitment of representatives with appropriate and explicit experience ensures that we fully understand the needs of our research participants.

This work builds on previous published work in PPI for trials and academic units [62, 63]. In line with the standards, the PPI representatives will be invited to join the Involving People network in order to benefit from its training portfolio and support systems. All usual arrangements, refreshments, travel, access and carer support will be in place for considerate inclusion of PPI representatives at meetings. The project will work to the NIHR’s newly developed National Standards for Public Involvement in Research and an audit of the standards will be reported at the close of the study [64].

## Trial status

This manuscript is a summary of the current, HTA-agreed protocol (Version 5 [17 August 2018]), with regard for NIHR dissemination guidance.

The study opened to recruitment in North Wales on 10 January 2018, on 1 April 2018 in Gloucestershire and on 21 March 2018 in Cardiff & Vale. Recruitment is aimed to be completed by the end of March 2019.

## Additional files

Additional file 1: Legal framework. (DOCX 23 kb)

## Abbreviations

AE: Adverse event
CI: Chief Investigator
CTIMP: Clinical Trial of an Investigational Medicinal Product
DCE: Discrete Choice Experiment
DMEC: Data Monitoring and Ethics Committee
DN: District nurse
GCP: Good Clinical Practice
GDPR: General Data Protection Regulation
GP: General practitioner
HCP: Healthcare professional
HRA: NHS Health Research Authority
HTA: Health Technology Assessment
IPA: Interpretative Phenomenological Analysis
ISRCTN: International Standard Randomised Controlled Trials Number
MHRA: Medicines and Healthcare products Regulatory Agency
MSAS-GDI: Memorial Symptom Assessment Score – General Distress Index
NICE: National Institute for Health and Care Excellence
NIHR: National Institute for Health Research
NWORTH: North Wales Organisation for Randomised Trials in Health
OOH: Out of hours
PI: Principal Investigator
PPI: Patient Public Involvement
QOLLTI-F: Quality of Life in Life Threatening Illness – Family Carer Version
RCT: Randomised control trial
REC: Research Ethics Committee
SAE: Serious adverse event
SC: Subcutaneous
SPC: Specialist Palliative Care
TMG: Trial Management Group
TSC: Trial Steering Committee
