# Supplementary material for: CARer-ADministration of as-needed subcutaneous medication for breakthrough symptoms in homebased dying patients (CARiAD): study protocol for a UK-based open randomised pilot trial
Source: Trials. 2019 Feb 7;20:105. doi: 10.1186/s13063-019-3179-9 (PMC6367805; doi:10.1186/s13063-019-3179-9)
Supplement: Supplementary file 1 — Legal framework. (DOCX 23 kb) [file 13063_2019_3179_MOESM1_ESM.docx]

# Legal framework

Clarity on legal issues is a significant aspect of this research to ensure lay carers and clinicians alike have legal protection. Our Australian partners have given us full access to their reference resource.[1] Their document covers a broad range of topics on the handling of medication in community-based palliative care services. It covers medication management, drug storage (security of medications, responsibility for medication storage, disposal of medication), prescribing, and medication administration (who can administer, record of administration) in the context of lay carer administration.

The premise:

A lay carer can legally administer medication individually prescribed for a third party, including controlled drugs such as morphine, as long as the carer has been appropriately trained and assessed as competent, specifically in medication management. This is true even if the medication is given to a patient lacking capacity, and/or if the medication is administered via injection. At present, injections are prepared immediately before administration (and not in advance, requiring relabelling). Carers should be trained to assess symptoms and should have access to dedicated support.

In support of these statements, the relevant sections from UK legislation and guidance are detailed below:

**A lay carer can administer medication individually prescribed for a third party, including controlled drugs such as morphine,**

- Section 7(3) of the Misuse of Drugs Regulations 2001 states: “*Any person other than a doctor or dentist may administer to a patient, in accordance with the directions of a doctor or dentist, any drug specified in Schedule 2, 3 or 4.*”[2]
- This was confirmed by the UK Medical Defence Union.[3]
- NHS NPC guidance (2009) states: “*A carer/relative can, with consent, administer a controlled drug (CD) that has been individually prescribed for a third party. As CDs are included within the legal category of prescription-only medicines (POMs), home carers who are competent to administer medicines should also be competent to administer CDs*”.[4]
- Morphine is listed in Schedule 2, and Midazolam in Schedule 3.[5]

**As long as the carer has been appropriately trained and assessed as competent,**

- Nursing & Midwifery Council (NMC) guidance, Standard 17: Delegation states: “*A registrant is responsible for the delegation of any aspects of the administration of medicinal products and they are accountable to ensure that the patient, carer or care assistant is competent to carry out the task. This will require education, training and assessment of the patient, carer or care assistant and further support if necessary. The competence of the person to whom the task has been delegated should be assessed and reviewed periodically. Records of the training received and outcome of any assessment should be clearly made and be available.*”[6]
- Department of Health, Social Services and Public Safety (Northern Ireland) guidance states: “*home carers who are appropriately trained and assessed as competent are authorised to administer orally prescribed controlled drugs*”[7]
- The Durham and Tess Valley Regional Medication Policy Group states: “*The cornerstone of the policy is a risk assessment to identify appropriate support for service users and the provision of appropriate training for those staff that will assist service users with medication. A carer administering a medicine will not be held responsible for any adverse effects, providing a medicine has been given in accordance with a prescriber’s instructions and local policies have been followed. Employing organisations should include medication tasks in any indemnity insurance they arrange.”*[8]

**Specifically in medication management.**

- The Durham and Tess Valley Regional Medication Policy Group continues: *“Carers will operate within a safe system which will be based on a risk assessment and this will need to be underpinned by a structured programme of education and learning in the safe handling, administration and management of medication.”*[8]
- Procedures are already in place in the UK to handle/store medications (including for anticipatory care purposes) in the patient’s home.[9]

**This is true even if the medication is given to a patient lacking capacity,**

- Medication can be given to a patient who lacks capacity if it is in his or her best interests. The Mental Capacity Act (MCA) 2005 Section 1(5) states: “*An act done, or decision made, under this Act for or on behalf of a person who lacks capacity must be done, or made, in his best interests*.”[10]
- The MCA 2005 permits the relevant actions to be performed by those with appropriate skills or expertise (as long as the carer has been appropriately trained and assessed as competent). The Code of Practice explains: “*To receive protection from liability under section 5, all actions must be related to the care or treatment of the person who lacks capacity to consent. Before taking action, carers must first reasonably believe that:*
  - *the person lacks the capacity to make that particular decision at the time it needs to be made, and*
  - *the action is in the person’s best interests.*”[11]

**And/or if the medication is administered via injection.**

- The Durham and Tess Valley Regional Medication Policy Group states that for specialist tasks (including injections) a suitable health professional needs to give additional training and confirm that the carer is competent to provide such care.[8]

**At present, injections are prepared immediately before administration**

- NMC Guidance, standard 14 states: “*Registrants must not prepare substances for injection in advance of their immediate use or administer medication drawn into a syringe or container by another practitioner when not in their presence*.”[6] The guidance continues: “*Where a registrant has delegated to a named individual for a named patient’s medication, this may be drawn up in advance to enable the healthcare assistant (HCA) or family to administer the medication. The registrant is accountable for the delegation, and a full risk assessment should be documented in the patient’s records ensuring the registrant is aware of the risks before agreeing to delegate.*”
- Note: There is evidence that the practice of drawing up and leaving these medications in syringes, for this type of practice, is safe in terms of sterility, potency and stability.[12] The team tested a full range of medications for 28 days.

**Carers should be trained to assess symptoms, use the least invasive methods of administration and should have access to dedicated support.**

# References

1. Brisbane South Palliative Care Collaborative. Guidelines for Handling of Medication in Community Based Palliative Care Services in Queensland. http://www.health.qld.gov.au/cpcre/pdf/medguidepall.pdf. Accessed 16 November 2018.

2. Legislation.gov.uk. Misuse of Drugs Act 1971 (Section 7). http://www.legislation.gov.uk/ukpga/1971/38/section/7. Accessed 16 November 2018.

3. Medical Defence Union. Reply to a request for information to Dr Lucy Boyland re: Is it legal for carers to administer controlled drugs? 2009.

4. NHS National Prescribing Centre. A guide to good practice in the management of controlled drugs in primary care (England); 2009.

5. British National Formulary. Controlled Drugs and drug dependence. http://www.evidence.nhs.uk/formulary/bnf/current/guidance-on-prescribing/controlled-drugs-and-drug-dependence . Accessed 23 November 2015.

6. Nursing & Midwifery Council. Standards for medicines management. http://www.nmc.org.uk/globalassets/siteDocuments/NMC-Publications/NMC-Standards-for-medicines-management.pdf. Accessed 16 November 2018.

7. Department of Health, Social Services and Public Safety. Safer Management of Controlled Drugs: A guide to good practice in primary care (Northern Ireland); 2013.

8. Durham and Tess Valley Regional Medication Policy Group. Model of Good Practice for the Development of Policy for the Safe Handling, Management and Administration of Medication by Carers within Domiciliary Care across the North East of England; 2008.

9. British Medical Association. GP practices: Focus on anticipatory prescribing for end-of-life care. http://bma.org.uk/support-at-work/gp-practices/service-provision/prescribing/focus-on-anticipatory-prescribing-for-end-of-life-care. Accessed 16 November 2018.

10. Legislation.gov.uk. Mental Capacity Act 2005 (Section 1). http://www.legislation.gov.uk/ukpga/2005/9/section/1. Accessed 16 November 2018.

11. Department for Constitutional Affairs. Mental Capacity Act 2005 Code of Practice. London; 2007.

12. Anderson B, Kralik D. Sterility, stability and potency of medications administered by carers in home-based palliative care setting. Research Informed Practice. 2008;51.
